# Prevalence and Risk Factors of Vulvovaginal Candidosis during Pregnancy: A Review

**DOI:** 10.1155/2022/6195712

**Published:** 2022-07-20

**Authors:** Tasfia Disha, Fahim Haque

**Affiliations:** ^1^Biotechnology Program, Department of Mathematics and Natural Sciences, BRAC University, 66 Mohakhali, Dhaka 1212, Bangladesh; ^2^Microbiology Program, Department of Mathematics and Natural Sciences, BRAC University, 66 Mohakhali, Dhaka 1212, Bangladesh

## Abstract

Vulvovaginal candidosis (VVC) is a symptomatic vaginal yeast infection, especially caused by *Candida* spp. Although VVC is common among reproductive-age women, prevalence studies notice the uprise of vaginal Candida colonization to 30% during pregnancy by culture, especially in the last trimester. Recent studies have considered it a severe problem due to the emerging evidence showing the association of VVC with a higher chance of pregnancy-related complexities (e.g., preterm labor, premature rupture of membranes, congenital cutaneous candidosis, and chorioamnionitis). In this review, we have reassessed and summarized the prevalence rate of VVC in expecting mothers and analyzed the association of several factors to the increased risk of VVC during pregnancy in different regions of the world. Altogether, these data collected from various studies showed the highest prevalence of VVC during pregnancy, mostly in Asian and African countries (90.38%, 62.2%, and 61.5% in Kenya, Nigeria, and Yemen, respectively). The prevalence rate of VVC during pregnancy was also found to differ with age, gestation period, parity, educational status, and socioeconomic level. Some pregnancy-related factors (e.g., weakened immunity; elevated level of sex hormones, glycogen deposition; low vaginal pH; decreased cell-mediated immunity) and several clinical and behavioral factors can be suggested as potential risk factors of candidosis during pregnancy.

## 1. Introduction

Candida infections in the vaginal area are frequently referred to as “Vulvovaginal candidosis” (VVC) or “Candida vaginitis.” Infection of the estrogenized vagina and the vestibulum that can spread to the outside of the labia minora, the labia majora, and the intercrural region is defined as vulvovaginal candidosis [1]. After bacterial vaginosis, it is considered the 2^nd^ most common among many causes of vaginitis [2]. It is produced most often by the overabundance of an opportunistic pathogenic yeast, *Candida albicans* (approximately 90%), which is a common member of the vaginal flora [3, 4]. This is a dimorphic commensal yeast usually involved in the colonization of the skin and reproductive and gastrointestinal tracts [2]. Almost 20 to 30% of healthy asymptomatic women may have this yeast within their vaginal tracts at any moment in their lifetime, if tested by culture, but more than 60%, if tested by NAAT methods [1, 5]. *Candida* spp. can cause an infection like VVC when the balance between the host and colonizing yeast gets temporarily disturbed. However, non-*albicans Candida* (NAC) species such as *glabrata*, *parapsilosis*, and *tropicalis* are also emerging as identifiable causes of VVC [3].

On the basis of episodic frequency, candida vaginitis can be either sporadic or recurrent [6]. Uncomplicated or sporadic VVC includes mild to moderate clinical signs and symptoms such as a thick cottage cheese-like discharge, pain, vaginal and vulvar pruritus, erythema, burning, and/or edema, along with external dyspareunia and dysuria [3]. Complicated or recurrent VVC may be defined as that which has recurrent episodes (4 or more episodes in a 12-month period) associated with severe symptoms [3, 6].

Around 75% of all women during their childbearing years experience at least one episode of VVC and about half among them have at least one recurrence [4, 7]. Generally, vaginal colonization of *Candida* species occurs in a minimum of 20% of all women which rises up to 30% in pregnancy [1]. During pregnancy, vulvovaginal candidosis is considered more common and difficult to eradicate because several normal and expected physiological changes in the genitourinary tract favor the growth of Candida [6, 8].

Some evidence in recent days shows the association of candidosis with an elevated risk of complications during pregnancy, like premature rupture of membranes and poor pregnancy outcomes including chorioamnionitis and preterm labor whereas congenital cutaneous infections are reported since decades as rare events during pregnancy [9, 10]. According to the literature, approximately 10–50% is considered to be the incidence of vaginal colonization with *Candida* species in pregnant women [11] and it is a significant problem as pregnant women can even contaminate their infants from 25% up to 65% which will result in invasive neonatal candidosis [12, 13]. Evidence showed that women with untreated asymptomatic candidosis had a greater spontaneous preterm birth rate compared to those who did not have candidosis (6.25 versus 2.99%) [14].

Susceptibility to VVC is enhanced by multiple risk factors, for instance, pregnancy, immunosuppression, HIV infection, diabetes, contraceptives, and antibiotic use [15, 16]. In addition, some pregnancy-related factors such as increased estrogen levels, increased vaginal mucosal glycogen production, and decreased cell-mediated immunity are likely to cause both asymptomatic colonization and the increased risk of VVC during pregnancy [15].

In this review, we have aimed to compile the recent data regarding the prevalence and risk factors of VVC during pregnancy. This review study has the following purposes: (1) to review previous papers on the prevalence of VVC, (2) to reassess and summarize the associated factors with VVC during pregnancy, and (3) to give an overview on the differences in prevalence and factors of VVC found out among pregnant women in different regions of the world.

## 2. Prevalence of Vulvovaginal Candidosis during Pregnancy

### 2.1. Higher Prevalence of VVC during Pregnancy

**Figure 1:**
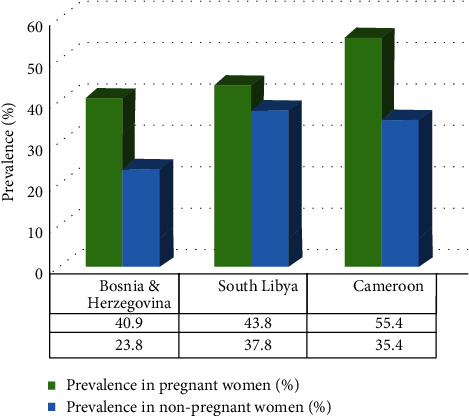
Difference in the prevalence rate of vulvovaginal candidosis (VVC) between studied nonpregnant and pregnant women. Data was collected from 3 randomly selected comparative literature [17–19]. Higher prevalence of VVC among pregnant women was observed.

Multiple studies have carried out a comparative study between nonpregnant and pregnant women and found out that pregnant women have a higher prevalence rate of VVC compared to nonpregnant women (Figure 1). Such a study was by Babić and Hukić where positive microscopic findings were remarkably increased in pregnant women samples, 40.9% (83/203) than in nonpregnant women samples, 23.8% (58/244) [17]. We noticed a similar type of results in South Libya with 43.8% [18] and in Cameroon with 55.4% prevalence [19] (Figure 1).

Grigoriou et al. performed the same type of study consistent with this result where the prevalence of VVC among 3,791 nonpregnant and 952 pregnant women with signs and symptoms of vaginitis was tested, and *Candida* spp. were detected in 299 (7.9%) nonpregnant women and 277 (29.1%) pregnant women [20]. Thus, these results highlight the higher prevalence of VVC during pregnancy.

### 2.2. Region-Wise Total Prevalence of VVC and/or Vaginal Candida Colonization among Pregnant Women

Our analysis found that the prevalence of VVC in pregnant women differs from region to region. In the USA, the prevalence of moderate to heavy levels of vaginal candidosis among pregnant women is estimated to be 10% [21]. The prevalence of vulvovaginal candidosis and/or vaginal candida colonization during pregnancy has been observed to be 17-90% in our analysis (Table 1).

Data has been organized alphabetically; collected from 27 published reports on the prevalence of VVC in pregnant women worldwide. VVC: vulvovaginal candidosis.

According to the data in Table 1, the highest prevalence of VVC to be noticed is 90.38% in Kenya, a study conducted by Nelson et al. [31]. Also, a significant prevalence of VVC with 62.2%, 60.8% positive for candidiasis, and 61.5% positive growth in culture has been observed in Enugu State, Nigeria [26]; North-west Nigeria [37]; and Ibb, Yemen [28], respectively. On the contrary, a study performed by Masri et al. [39] in Malaysia has detected the lowest positive isolates for candida, which is 17.2% during pregnancy. The widespread moderate to high level of prevalence of VVC among childbearing women can be perceived through this data (see Table 1).

The differences in the prevalence rates across the world can be because of geographic, ethnic, and socioeconomic factors and varying sampling and culturing techniques [29].

### 2.3. Comparison between the Prevalence Rate of VVC and Asymptomatic Vaginal Candida Colonization

Vulvovaginal candidosis incorporates the range of patients who have tested positive for cultures of *Candida* spp. with symptoms like having an appearance of florid, severe disease [6]. The prevalence rate of VVC and asymptomatic colonization during pregnancy is shown in Table 2.

Data has been collected from 11 literature and arranged alphabetically.

Several studies found a higher prevalence of symptomatic infection caused by *Candida* species during pregnancy. Table 2 shows that majority of the places had detected a higher prevalence of symptomatic VVC. Among them, the highest symptomatic prevalence was reported in Sana'a, Yemen, that is 86.2% [38]. Edrees et al. [28] and Ghaddar et al. [32] had also found a higher prevalence of *Candida* (+) spp. among pregnant women in Ibb, Yemen, and Lebanon with 61.2% and 82%, respectively. Along with that, similar results were found out in Mato Grosso, Brazil [34], Tunisia [43], and North-west Nigeria [37] where a majority had expressed signs and symptoms of VVC.

Yet, other studies showed that asymptomatic Candida infections were more probable to occur during pregnancy. For example, Mucci et al. [24] had found 61.9% asymptomatic colonization among pregnant women in Argentina (Table 2). Furthermore, 67.9%, 68.2%, and 70.74% asymptomatic *Candida* (+) spp. were detected in Kathmandu, Nepal [30], Middle Belt of Ghana [35], and Burkina Faso [25], respectively. These results were consistent with the previous one. Thus, these results justify the higher incidence of asymptomatic VVC in pregnant women. On the other hand, Okonkwo and Umeanaeto [22] found no significant difference in the prevalence between symptomatic and asymptomatic pregnant women.

### 2.4. Prevalence of Different Species of Candida among Pregnant Women

In a study, *Candida albicans* has been isolated from more than 80% of specimens obtained from women with vulvovaginal candidosis and has been considered the most common causative yeast for VVC [44]. A 5 yearlong epidemiological survey on the causative agents of VVC had also found *Candida albicans* as the most prevalent cause in 87.9% of cases [45]. The species that have been mostly identified in several studies are *C. albicans*, *C. glabrata*, *C. krusei*, *C. tropicalis*, *C. lypolytica*, *C. kefyr*, *C. famata*, *C. parapsilosis*, and *C. dubliniensis*. In Table 3, the prevalence of *C. albicans* and NAC spp. isolated from pregnant women around many parts of the world has been presented. It also manifests the dominance of *C. albicans* over other non-*albicans Candida* spp. in the majority of the cases. The highest prevalence of *C. albicans* that had been recorded from the samples of pregnant women was 95% in Natal, Brazil [36]. Dias et al. [34] also had found a prevalence of 92.3% for *C. albicans* in Mato Grosso, Brazil. Some other areas like Sarajevo, Bosnia and Herzegovina [17], Malaysia [39], and Argentina [24] had detected a significant prevalence of *C. albicans*, which was 87.4%, 83.5%, and 80.7%, respectively.

*C. glabrata* is considered the most prevalent species among other NAC spp. to be associated with VVC by Ghaddar et al. [32] which is consistent with the data mentioned in Table 3. On the other hand, Edrees et al. [28] showed a higher prevalence of *C. tropicalis* (21.64%) over *C. glabrata* (11.19%). A study by Tsega and Mekonnen [16] also showed a similar type of result where *C. krusei* (21.9%) was higher than *C. glabrata* (17.7%). Ahmad and Khan [46] had found *C. parapsilosis* to be the second most common non-*albicans Candida* species following *C. glabrata*. According to our study, the most commonly detected non*-albicans candida* spp. after *C. glabrata* were *C. krusei* and *C. tropicalis* (see Table 3).

**Figure 2:**
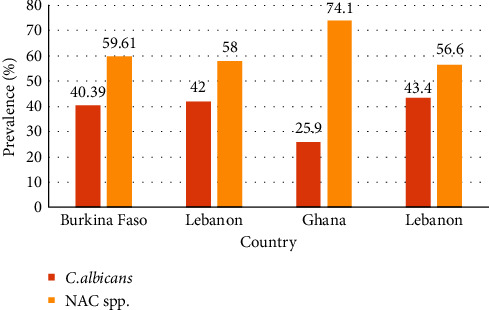
Difference in the prevalence rates of *Candida albicans* and non-*albicans Candida* spp. in Asian and African countries. Here, dominance of non-*albicans Candida* spp. over *Candida albicans* has been observed.

Although Candida albicans is found accountable for most of the vaginal candidosis-related symptoms in numerous studies worldwide, multiple Asian and African countries in the past three decades have observed a noticeable uprise in the detection rate of non-albicans *Candida* spp. together with infections caused by them [17, 29]. Multiple studies have found a higher occurrence of non-albicans *Candida* spp. over Candida albicans (see Figure 2). Waikhom et al. [27] found the highest cumulative prevalence of NAC spp., 74.1%, where *C. glabrata* alone accounted for 57.4% of prevalence among pregnant women in Ho municipality, Ghana. Ghaddar et al. [33] and Sangaré et al. [25] also found 58% and 59.61% prevalence of NAC spp., respectively. Another study by Ghaddar et al. is where 56.6% (*C. glabrata* 44.5%) prevalence of NAC spp. was observed in Lebanon [32]; this result correlated with the previously mentioned ones (see Figure 2). The outcome of studies concerning the frequency of vulvovaginal candidosis indicates that the isolated Candida species distribution among pregnant women varies from country to country, and risk factors like age, hygienic habits, and disease history have a remarkable influence on that [32].

### 2.5. Age Group-Wise Distribution of VVC

Several studies had divided their studied pregnant women population into different age groups to identify the age group with the highest prevalence rate of VVC in those respective studies (see Table 4).

Data has been arranged alphabetically. The age groups with the highest prevalence in respective studies are in bold form. Abbreviation: VVC: vulvovaginal candidosis.

The range of age groups with the highest occurrence of VVC in different studies is between 16 and 37 years. In our study, the highest prevalence of VVC has been noticed in the age group 25–29 years with 68.3% [42] (Table 4). The other age groups of pregnant women with almost relative prevalence are 26–30 years [40], 20–24 years [38], and 26–35 years [31].

There is no any common single age group constantly with significant prevalence among different studies; rather it varies from region to region. The possible reasons might be the relation of these results to the fact that participants of the respective groups use drugs and contraceptives indiscriminately to prevent pregnancy [18] and are also highly sexually active [32]. Another reason could be that the association of age group with the prevalence of VVC was affected by other variables [38].

### 2.6. Gestational Period–Wise Distribution of VVC

Multiple studies had identified the gestation period in which the frequency of VVC was the highest in that respective study to find out any correlation between the gestation period and the prevalence of VVC during pregnancy.  Table 5 shows the data collected from those studies.

Data has been collected from previously reported literature and organized alphabetically; the gestational period with the highest prevalence is in bold form. VVC: vulvovaginal candidosis.

According to Kinghorn [47], the prevalence of VVC increases with the progression of pregnancy, especially in the last trimester. The majority of the prevalence mentioned in Table 5 is consistent with this, where most of the highest prevalence had been observed in the 3^rd^ trimester. Among all of them, a study by Olowe et al. [41] had found the highest (93%) prevalence of VVC in the last trimester of pregnancy. In contrast, the first and second trimesters also had shown the highest occurrence of VVC in a couple of studies (Table 5). In the case of the gestation period, this difference in rates of VVC could be due to the difference in sample size and study participants [16]. Therefore, it cannot be firmly said that prevalence increases only with the gestational period as women in their 1^st^ and 2^nd^ trimester also had shown a higher risk of getting vulvovaginal candidosis [39]. On the other side, Tsega and Mekonnen [16] did not find any significant differences between the gestational period and *Candida* colonization.

### 2.7. Socioeconomic Level, Educational Status, and Parity-Wise Distribution of VVC

In 2020, Edrees et al. [28] and Al-Rukeimi et al. [38] had found the highest prevalence of VVC among pregnant women from rural areas (65%) and low socioeconomic levels (60.4%), respectively.

**Figure 3:**
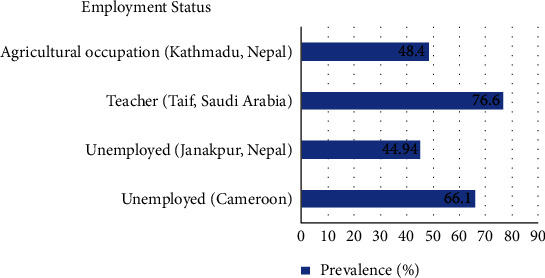
Prevalence of VVC according to employment status in different countries. Abbreviation: VVC: vulvovaginal candidosis.

Toua et al. had found out highest prevalence in the unemployed (66.1%) group of pregnant women [19] (Figure 3). Similarly, unemployed women showed the highest prevalence of 44.94% in a study by Yadav and Prakash [29] whereas the other two studies by Shrestha et al. [30] and Al-Aali [42] had shown the highest prevalence in women from the agricultural (48.4%) and teacher (76.6%) occupation, respectively (Figure 3).

**Figure 4:**
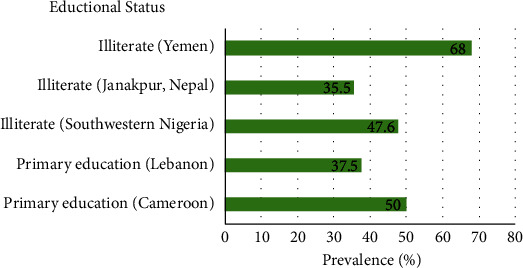
The difference in the prevalence rates of VVC according to educational status. Abbreviation: VVC: vulvovaginal candidosis.

In a study by Al-Rukeimi et al. [38], illiterate women showed the highest prevalence (68%) in Sana'a, Yemen, whereas patients with university education had a 39.7% prevalence. In Janakpur, Nepal [29], and Southwestern Nigeria [41], pregnant women with no education had 35.5% and 47.6% prevalence, respectively. On the other hand, pregnant women with primary education had been observed to show the highest prevalence (37.5%) in Lebanon [32]. A similar result was found in Maroua, Cameroon, with a 50% prevalence in the group with primary education [19] (see Figure 4). The difference in the prevalence rate among the illiterate and the educated may be explained by the improvement in personal hygiene and/or the economic situation resulting from education [38].

However, the prevalence rate of VVC showed no noteworthy difference between highly educated women and women with tertiary education [22].

**Figure 5:**
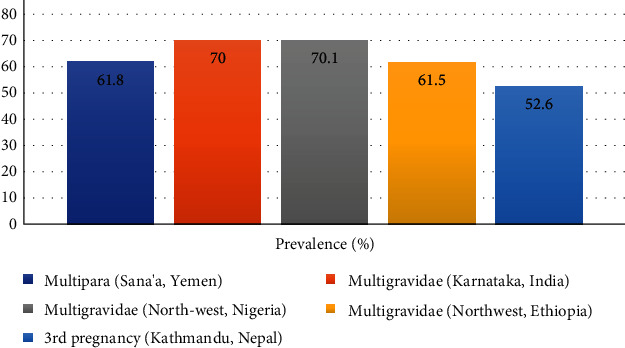
Parity- and gravida-wise prevalence of VVC in pregnant women of different regions. Abbreviation: VVC: vulvovaginal candidosis.

Multigravidae (61.5%) mothers are found to have a high rate of *Candida* colonization compared to primigravidae (38.5%) [16]. Nnadi and Singh [37] and Kanagal [40] also had found the highest prevalence of VVC that is 70.1% and 70%, respectively, in multigravidae women. Women with multipara of parity had a 61.8% prevalence which was higher than the prevalence in pauciparous (54.4%) or nulliparous women (38.5%) [38]. In Kathmandu, Nepal women in their 3^rd^ pregnancy had the highest prevalence (52.6%) among the study participants; this result is consistent with the previous results [30] (Figure 5). But, contrariwise, Okonkwo and Umeanaeto [22] found out that the proportion of VVC decreased with parity. The described possible reason for this was the women's experience relating to pregnancy and infections giving birth to multiple babies.

## 3. Risk Factors of VVC during Pregnancy

Pregnancy is regarded as a risk factor because of the over-sensitivity of the vagina during that time, facilitating infections to occur more frequently [29]. The role of pregnancy has been correlated positively with the occurrence of VVC [43]. The expression of symptomatic VVC amid pregnancy is dependent on some demographical, clinical, and behavioral factors [48]. Some host-related factors such as genetic predisposition, uncontrolled DM, behavioral factors (e.g., antibiotic use, contraceptive use), and conditions with high reproductive hormone levels during pregnancy have also been described to be associated with VVC [49, 50].

### 3.1. Pregnancy-Related Factors

During pregnancy, several physiologic change-related factors in pregnant women including the weakened immune system, increased level of reproductive hormones, glycogen deposition, low vaginal pH, and decreased cell-mediated immunity have been addressed in literature as risk factors of VVC.

#### 3.1.1. Weakened Immune System

The impaired immune system makes pregnant women more susceptible to infections [32]. Excess stress had been described as a possible reason for this [23, 42]. Emotional stress increase as a woman is expecting a child, which results in the suppression of the immune system. The weakened immune system ultimately steps up the overgrowth of Candida spp. and becomes pathogenic [24, 27].

#### 3.1.2. Increased Level of Reproductive Hormones

With the progression of pregnancy, hormone levels fluctuate drastically and become notably greater than normal time [51]. During pregnancy, elevated secretion of sex hormones, both progesterone and estrogen had been found to favor the formation of infection [29]. A high level of progesterone allows the Candida yeast to implant in the vagina by causing an alteration in the vaginal epithelium [43]. In addition, progesterone possesses inhibitory effects on the anti-candida activity of neutrophils [29]. The healthy balance of microorganisms can get upset by the increased estrogen level, which in return enhances the possibility of vaginal candidosis establishment [28]. High levels of estrogen have been found to facilitate the attachment of yeast to mucosal epithelial cells of the vagina [4]. Along with that, estrogen stimulates growth, multiplication, hyphal formation [11], and enzyme elaboration for instance secreted aspartyl proteinase and phospholipases which increase colonization [15]. Moreover, a high level of estrogen has been found to reduce immunoglobulins in vaginal secretions and decrease the epithelial cells' ability to suppress the growth of Candida albicans leading to the increased vulnerability to vaginitis during pregnancy [29].

#### 3.1.3. High Amount of Glycogen Deposition

Both progesterone and estrogen contribute to the elevation of vaginal tissue glycogen content [17, 43]. This high level of glycogen deposition provides an adequate source of carbon, thus favoring the growth and germination of Candida spp. on the wall of the vagina [17]. Hence, it may be responsible for the increased susceptibility of pregnant women to VVC by giving a favorable room for Candida enhancement.

#### 3.1.4. Decreased Level of pH

Typically, the vaginal pH is maintained at 4.0-4.5, and this level of acidic environment prevents the establishment of many vaginal pathogens [22]. Yadav and Prakash [29] stated that any physiological change affecting both beneficial and harmful vaginal microorganisms alters the acidity of the vagina that reduces its pH to 5.0-6.5; this would thereby enhance the establishment of pathogenic organisms such as *Candida.* Increased level of progesterone during pregnancy has been shown to decrease the vaginal pH, thus favoring a suitable environment for *Candida* yeast overgrowth [43].

#### 3.1.5. Decreased Cell-Mediated Immunity

During pregnancy, the immunologic changes might have a role in the alteration of severity and susceptibility to infections at that time. The immune system and reproductive hormones have a multifactorial and complex interplay between them. Cell-mediated immunity is important during pregnancy for altered responses to infections [51]. Estrogen-enriched states as in the last trimester of pregnancy have been involved in the suppression of the cell-mediated immunity [52, 53] . Progesterone has been found to change the balance between Th1 and Th2 responses [15] and suppress the maternal immune response [54, 55]. As gestation advances, estradiol levels can rise as much as 500-fold in the maternal serum [56] and high estradiol concentrations are involved in the augmentation of humoral immunity as well as CD4+ type 2 helper T-cell (Th2) responses [57]. Along with that, levels of cytokines increase during pregnancy which stimulates phagocytic cell recruitment or activity [56]. A current approach had suggested a switch from Th1 to Th2 immunity in the time of pregnancy [58]. Th2 cells decrease cell-mediated immunity by inducing B lymphocytes, increasing antibody production, and suppressing the cytotoxic T-lymphocyte response [51].

Existing evidence claims that features of innate immunity (phagocytic activity, levels of *α*-defensin, and monocytes, neutrophils, and dendritic cell numbers) are increased with the progression of pregnancy, especially in the second and third trimester. In contrast, CD3+ T lymphocytes (both CD4+ and CD8+) count in the blood is decreased [59]. Antifungal responses throughout pregnancy can be affected by this decrease in the numbers and activity of CD4+ cells, CD8+ cells, T-cells, and natural killer cells and as a result slows down the removal of harmful microorganisms [51].

#### 3.1.6. Gestation Period

Several studies have linked the trimester of pregnancy with the vulnerability of pregnant women to VVC. The vulnerability of pregnant mothers to infection increases with the progression of pregnancy, hence the highest prevalence in the third trimester [22]. According to Nelson et al. [31], an increased estrogen level and corticoids in the 3^rd^ trimester decrease the vaginal defense mechanism against such opportunistic fungus. Along with that, the repetitive vaginal and pelvic examination, reduction in hygiene statuses such as failure to wash undies and pelvic areas due to fatigue or the tummy size of the pregnant mothers could encourage vaginal infection and predispose them to greater chances of VVC in the last trimester of pregnancy [22]. Guzel et al. [11] had found an increase in the prevalence with gestation week, which is consistent with the previously mentioned reasons, whereas Masri et al. [39] found pregnant women in their 1^st^ and 2^nd^ trimester to a higher risk of getting VVC which is contradictory. However, the third trimester of pregnancy had been statistically insignificant, with a higher occurrence of VVC in multiple studies [25, 27, 29, 37]. Waikhom et al. [27] and Yadav and Prakash [29] had excluded pregnant women with any complications such as diabetes, previous preterm labor, and those who were on antibiotics which could be a possible reason for finding no relation between VVC and gestational period. So, the role of the gestational period, especially the last trimester as a risk factor for vulvovaginal candidosis during pregnancy, is still controversial.

### 3.2. Clinical Factors

Diabetes mellitus, HIV infection, and previous encounters with candidosis have been discussed in several studies as potential factors contributing to vaginal colonization during pregnancy.

#### 3.2.1. Diabetes Mellitus

Uncontrolled diabetes acts as a predisposing factor to VVC [43]. Patients with clinical diabetes mellitus have an increased risk of *Candida* infections of the skin and vagina [60]. In diabetes mellitus, glucose concentrations get increased in the vaginal secretions [47] which stimulates adherence of *Candida* to epithelial cells and promotes its development and effective expression of virulence factors [43]. The capability of eliminating pathogen by neutrophils and also phagocytosis is limited by hyperglycemia condition [43]. In addition, hyperglycemia can stimulate protein production in *Candida* spp., which facilitates yeast adherence as well as destroys phagocytosis by the host [61]. Hence, pregnant women with diabetes may be more prone to VVC as it enhances the growth of yeasts. A statistically significant association between diabetes and the rate of VVC during pregnancy had been found in a study by Masri et al. [39] which follows the result found by Kanagal [40]. *C. albicans* was found to be significantly associated with gestational diabetes [33]. Nevertheless, several studies did not find any significant statistical relationship between diabetes and VVC during pregnancy [29, 36, 43]. Guzel et al. [11] also found no association of DM with the prevalence of candida vaginitis during pregnancy. The possible reason for this disassociation could be the susceptibility of diabetic-positive pregnant women to other infections such as bacterial vaginosis, resulting in the reduced risk of VVC because of the genesis of bacterial toxins and competition for available sources of micro-nutrients, energy resources, and mucosal binding sites [36].

#### 3.2.2. HIV Infection

Immunocompromised women are generally at increased risk of fungal infections. It has been shown in studies conducted earlier where increased vaginal colonization with fungi has been caused by a loss of immune-protective mechanisms [62]. In immunosuppressed patients, vaginal candidosis can be correlated well with reduced cell-mediated immunity [43]. Predisposing host factors, such as HIV infection and other immunosuppressive diseases, play the leading role in the development of VVC. Moreover, proteinase activity acts as a key role in the pathogenesis of VVC, which gets increased in HIV-positive women, hence makes them susceptible to VVC [62]. Yet, in several studies, there was a statistically insignificant association between HIV infection and VVC [16, 29, 43] whereas Foessleitner et al. [62] found out greater than a twofold increased risk of VVC in HIV-positive pregnant women compared with the HIV-negative control group. One probable cause of finding an insignificant relationship may be the fact that severely immunocompromised patients, in particular, are more likely to develop VVC [43].

#### 3.2.3. Past Episodes of Candidosis

Patients with a previous history of candidosis have been considered at a greater risk of developing VVC during pregnancy by some authors. It might be due to the hormonal milieu and suppressed immune system which contributes to the increased susceptibility. During pregnancy, a large proportion of women with chronic recurrent candidosis are the ones to be the first present with the infection [29]. A statistically significant 60% of candida-positive pregnant women with previous candidosis was found in a study by Kanagal [40]. On the other hand, Yadav and Prakash [29] found patients with a past history of candidosis to be statistically insignificant with the occurrence of VVC. Hence, it is difficult to claim previous candidosis as a reliable risk factor for developing VVC.

### 3.3. Behavioral Factors

Several behavioral characteristics of pregnant women might affect the rate of candida colonization during pregnancy. Behavioral factors such as use of antibiotics, oral contraceptives, intrauterine devices; tight clothing; douching habits and poor personal hygiene, and poor dietary habits have been assessed as risk factors of VVC during pregnancy in several studies.

#### 3.3.1. Frequent Use of Oral Contraceptives

Pregnant women who have been using oral contraception are considered being at an increased risk of developing vulvovaginal candidosis. Oral contraceptives cause many changes in the vaginal environment that might be associated with the decreased ability to resist Candida infection. Usage of high-dose contraceptive pills (75-150 *μ*g of mestranol) has been observed to affect glucose resistance over a small period which may, in turn, promote Candida adhesion or virulence by affecting the carbohydrate source in the vaginal epithelial cells [61]. In addition, oral contraceptives are found to be associated with immunological changes, including the elevation of antibodies in cervical mucous and the sera [63, 64], and probably the depression of T- lymphocyte proliferation [52]. Furthermore, most oral contraceptives have been found to contain estrogen and progesterone, which creates an “estrogen dominance” by disrupting the hormonal balance that results in enhancing Candida growth [43]. A statistically significant association between the prevalence of VVC and previous use of oral contraceptives has been confirmed by two studies [16, 40]. Still, evidence on the risk of VVC in pregnant women using oral contraceptives is conflicting because several studies have found no significant correlation between the use of oral contraceptives and the prevalence of VVC [29, 35, 43]. This could be due to the use of low-dose [61] and low estrogen-containing [65] oral contraceptives, as they were not considered significant risk factors for VVC.

#### 3.3.2. Prolonged Use of Antibiotics

An expanded chance of developing symptomatic VVC in pregnant women following a course of oral antibiotics has been depicted [66]. Continuous and misuse of drugs lead to resistance towards drugs, particularly towards the common antifungal agents utilized for the treatment of vaginal candidosis [29]. Broad-spectrum antibiotic use (e.g., tetracycline, ampicillin, cephalosporin) is capable of eliminating *Lactobacillus* spp. present in the normal defensive bacterial flora of the vagina, which prevents germination of Candida by providing a colonization resistance mechanism [43]. Moreover, antibiotics may play a vital role in the overgrowth and increased virulence of the *Candida* spp. by decreasing the prevalence of other competitive bacterial organisms to Candida for the substrate [67]. However, published data on this risk factor are conflicting. Some authors have found prolonged antibiotic use to be significantly associated with the prevalence of VVC during pregnancy [16, 40, 41]. Others have shown that pregnant mothers who have a history of antibiotic use do not have an increased prevalence of VVC during pregnancy [29, 35–37, 43].

#### 3.3.3. Use of Intrauterine Device (IUD)

The intrauterine device (IUD) has been considered another determinant in the genesis of vaginal candidosis. It can be elucidated by the adhesion and production of biofilm of *C. albicans* on the surface of IUD, which contributes to colonization, reduction of antifungal susceptibility, and exhaust to the host immunity. In this way, it contributes to the occurrence of recurrent VVC [43]. Kanagal [40] found a highly significant association of IUD user pregnant mothers with the prevalence of VVC, whereas Mtibaa et al. [43] and Yadav and Prakash [29] found a statistically insignificant correlation between the use of IUD devices and the prevalence of vaginal candidosis.

#### 3.3.4. Tight and Synthetic Clothing

In literature, the types of undergarments and clothing that usually women wear have been proposed as a risk factor of vulvovaginal candidosis. Al-Aali [42] had mentioned that the overgrowth of Candida was enhanced by the use of tight nylon underwear. Increased temperature, moisture, or direct irritation of the vaginal area are considered the possible mechanisms related to this [61]. Wearing tight clothes and synthetic underwear appears to increase the local acidity by nourishing friction and maceration, hence increase the fungal infection [43]. Despite that, the role of this factor in the prevalence of VVC during pregnancy has still been unproven and anecdotal.

#### 3.3.5. Dietary Habits

The role of dietary habits in VVC has been suggested as a risk factor because of the altered virulence of Candida in response to the heightened availability of sugar substrates [68–71]. Altayyar et al. [18] had mentioned poor dietary habits as a cause of the higher prevalence of VVC among pregnant women. Patients with VVC were more likely to excrete sugars such as sucrose, arabinose, and ribose. The associated dietary patterns with these sugars were an elevated intake of milk, yogurt, cottage cheese, and artificial sweeteners. Reduction in both the rate of VVC and the presence of sugars in urine were reported by less dairy ingestion [72]. However, data showing strong relation of the diet with the prevalence of VVC during pregnancy are scarce. Maximum studies could not prove the role of excess or deficient diet in the etiology of sporadic or recurrent vulvovaginal candidosis [6].

#### 3.3.6. Douching Habits and Female Hygiene Products

The microbial flora of the vagina can be altered by frequent douching with antiseptics, thus exposing it to Candida infection [19]. Yet, studies have failed to find an association between the incidence of vulvovaginal candidosis and douching habits during pregnancy [61]. Olowe et al. [41] also had found no association between douching and VVC.

The presence of Candida organism in the vaginal area may get influenced by the types and frequency of use of sanitary products. The possible reasons making them susceptible to infection include direct irritation, drying of the mucosal barrier, mucosal tears, and sensitivity to components and perfumes in the products [61]. Still, there is not enough evidence showing that menstrual protection (e.g., sanitary napkins or tampons) usage increases the risk of vaginal candidosis among pregnant women [6, 73].

#### 3.3.7. Stress

The validity of hypothesis considering stress as the root cause of Candida albicans vulvovaginitis has been confirmed in many cases [74]. The reason can be explained by the difference between the demands of a woman's surroundings and her ability to cope with them which actually causes the stressors to occur. Women who have their psychological and physical capacities pushed to the maximum or even exceeded are subjected to these stressors. The patient's immune system gets physiologically attenuated as an impact of the stressors [74].

## 4. Conclusions

Women in their reproductive age experience at least one episode of candidosis. The rate of Candida colonization has been found to increase during pregnancy, particularly in the 3^rd^ trimester. It has become a matter of concern due to the emerging evidence on the association of VVC with increased risk of pregnancy-related complications, for example, premature delivery and low birth weight. In our analysis, the prevalence of VVC among pregnant women across the world varied from the lowest 17% to the highest 90%. Pregnant women in Asian and African countries have shown the highest prevalence of VVC. In this study, reviewed data identified *C. albicans* as the leading causative agent for VVC, followed by *C. glabrata*, *C. krusei*, and *C. tropicalis.* Prevalence studies have revealed that the rate of candidosis varies with age, parity, gestation period, and socio-demographic factors. Some studies have noticed a higher prevalence of symptomatic VVC among pregnant women, while others have found expression of asymptomatic VVC at a greater rate.

**Figure 6:**
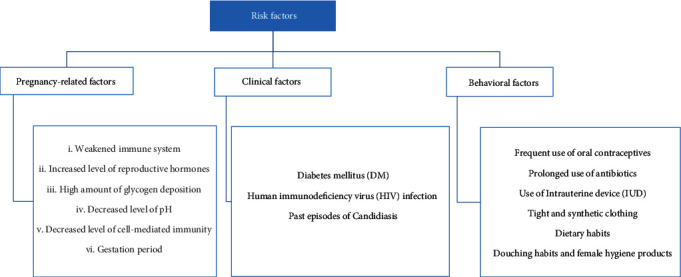
Compilation of different factors associated with VVC during pregnancy. Abbreviation: VVC: vulvovaginal candidosis.

In Figure 6, the risk factors of VVC during pregnancy have been summarized. Reviewed pieces of literature have assessed multiple pregnancy-related, clinical, and behavioral factors as risk factors for developing VVC during pregnancy, but not all have been found associated significantly with increased risk of VVC.

**Figure 7:**
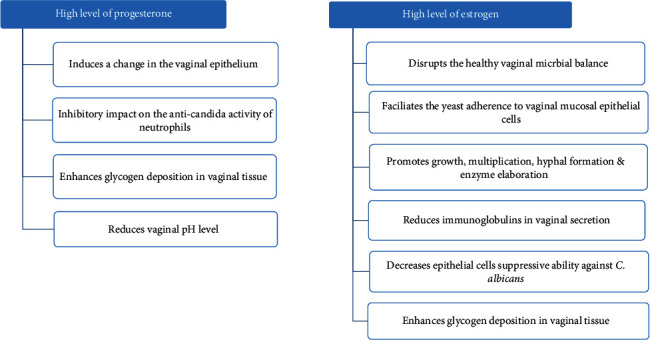
Summary of the consequence of the elevated level of progesterone and estrogen during pregnancy.

Increased level of reproductive hormones, especially estrogen and progesterone, has been found to significantly influence several physiological and immunological changes in pregnant women, which further favors Candida colonization in the vagina. The impact of elevated levels of sex hormones is summarized in Figure 7.

In the majority of the time, VVC is treated by observing clinical symptoms; hence, the data on the prevalence rate during pregnancy is not satisfying. Also, the role of associated factors with VVC is conflicting. Therefore, it can be said that studies on the prevalence rate and risk factors of vulvovaginal candidosis during pregnancy should be carried out more across the world, especially in third world countries to assess the actual scenario.

## Figures and Tables

**Table 1:** The prevalence of VVC and/or positive vaginal candida colonization during pregnancy in different regions of the world.

City, country	No. of studied patients	Prevalence (%)	Citations
Adana, Turkey	372	37.4	[11]
Anambra State, Nigeria	300	30	[22]
Ardabil, Iran	408	35	[23]
Argentina	210	24.8	[24]
Burkina Faso	229	22.71	[25]
Enugu State, Nigeria	901	62.2	[26]
Ghana	176	30.7	[27]
Ibb, Yemen	218	61.5	[28]
Janakpur, Nepal	157	35	[29]
Kathmandu, Nepal	200	29.5	[30]
Kenya	104	90.38	[31]
Lebanon	221	44.8	[32]
Lebanon	258	39	[33]
Maroua, Cameroon	112	55.4	[19]
Mato Grosso, Brazil	404	44.8	[34]
Middle Belt of Ghana	589	36.5	[35]
Natal, Brazil	41	48.78	[36]
Northwest Ethiopia	384	25	[16]
North-west Nigeria	288	60.8	[37]
Sana'a, Yemen	190	51.6	[38]
Sarajevo, Bosnia and Herzegovina	203	40.9	[17]
Selangor, Malaysia	1163	17.2	[39]
South Karnataka, India	118	42.37	[40]
South Libya	150	43.8	[18]
Southwestern Nigeria	100	36	[41]
Taif, Saudi Arabia	1207	70.2	[42]
Tunisia	2160	32.87	[43]

**Table 2:** The previously reported prevalence of VVC and asymptomatic vaginal candida colonization.

City, country	Asymptomatic vaginal candida colonization (%)	VVC (%)	Citations
Adana, Turkey	37.5	61.2	[11]
Argentina	61.9	38.1	[24]
Burkina Faso	70.74	29. 26	[25]
Enugu State, Nigeria	27	70	[26]
Ghana	18.5	81.5	[27]
Janakpur, Nepal	40.12	59.87	[29]
Kathmandu, Nepal	67.9	32.1	[30]
Middle Belt of Ghana	68.2	31.8	[35]
Northwest, Ethiopia	18.7	40.5	[16]
Sana'a, Yemen	22.3	86.2	[38]
South Karnataka, India	18	82	[40]

**Table 3:** The distribution of different species of Candida isolated from pregnant women across the world.

City, Country & Citations	NAC spp.								
	C. albicans (%)	C. glabrata (%)	C. krusei (%)	C. tropicalis (%)	C. lypolytica (%)	C. kefyr (%)	C. famata (%)	C. parapsilosis (%)	C. dubliniensis (%)
Adana, Turkey [11]	58.0	19	2.9	13.2		2.4	1.5	0.5	0.5
Argentina [24]	80.7	3.8							3.8
Burkina Faso [25]	40.39	32.69	11.54	15.38					
Ghana [27]	25.9	57.4	11.1					5.4	
Ibb, Yemen [28]	61.2	11.19		21.64		5.97			
Janakpur, Nepal [29]	64.04	12.35	3.37	5.61					10.11
Kenya [31]	63.83	29.79	2.13	3.19				1.06	
Lebanon [32]	43.4	44.5	12.1						
Lebanon [33]	42	41	17						
Malaysia [39]	83.5	16					0.05		
Mato Grosso, Brazil [34]	92.3	2.2	3.3	1.1				1.1	
Natal, Brazil [36]	95	5							
Northwest, Ethiopia [16]	56.25	17.7	21.96	1					
Sana'a, Yemen [38]	39.5	4.7			3.2		2.1		0.52
Sarajevo, Bosnia & Herzegovina [17]	87.4	4.2	3.2	2.1					
South Karnataka, India [40]	69.23	23.07		7.69					
Taif, Saudi Arabia [42]	70.2	16.5		3.3	2.6	0.6			
Tunisia [43]	76.61	17.18	1.54	1.4		0.56			

Abbreviations: NAC: non-*albicans Candida* and C. denotes *Candida.*

**Table 4:** Prevalence of VVC among pregnant women according to their age in different studies. The age groups with the highest prevalence in respective studies are in bold form. Abbreviation: VVC= vulvovaginal candidosis.

City, country	Age groups (years)	Prevalence (%)	Citations
Ho municipality, Ghana	<20	11.1	[27]
	**20 - 29**	**44.4**	
	30 - 39	40.7	
	40 - 49	3.7	

Ibb, Yemen	18 – 27	8.95	[28]
	**28 – 37**	**54.48**	
	38 – 47	36.57	

Janakpur, Nepal	15 – 20	19.10	[29]
	**21 – 25**	**40.44**	
	26 – 30	32.58	
	>30	7.86	

Karnataka, India	20 – 25	18	[40]
	**26 – 30**	**64**	
	30 – 35	12	
	>35	06	

Kenya	15 – 25	26	[31]
	**26 – 35**	**60**	
	36 – 45	12	
	>46	2	

Lebanon	20 – 25	12.5	[32]
	**26 – 30**	**34.1**	
	31 – 40	31	

Maroua, Cameroon	<18	4.8	[19]
	18 – 24	25.8	
	**25 – 31**	**46.8**	
	32 – 38	16.1	
	≥39	6.5	

Northwest Ethiopia	**18 – 25**	**50.5**	[16]
	26 – 33	42.7	
	34 – 40	6.8	

North-west Nigeria	16 – 20	8	[37]
	21 – 25	33.7	
	**26 – 30**	**37.1**	
	31 – 35	16	
	36 – 40	5.1	

Sana'a, Yemen	<20	33.3	[38]
	**20 – 24**	**61**	
	25 – 29	41.3	
	30 – 34	42.8	

South Libya	**16 – 25**	**46.7**	[18]
	26 – 35	40.7	
	36 – 45	36.1	

Southwestern Nigeria	21 – 25	14.3	[41]
	26 – 30	33.9	
	**31 – 35**	**46.9**	
	36 – 40	20	

Taif, Saudi Arabia	15 – 19	5.1	[42]
	20 – 24	6.6	
	**25 – 29**	**68.3**	
	30 – 34	9.6	
	35 – 39	7.8	
	40 – 44	3.4	

Data has been arranged alphabetically.

**Table 5:** Prevalence of VVC during pregnancy according to the no. of trimester; the gestational period with the highest prevalence is in bold form. VVC: vulvovaginal candidosis.

City, country	No. of trimester	Prevalence (%)	Citations
Burkina Faso	1^st^	22.78	[25]
	2^nd^	17.78	
	**3** ^**rd**^	**24.71**	

Ho municipality, Ghana	1^st^	16.7	[27]
	2^nd^	25.9	
	**3** ^**rd**^	**57.4**	

Janakpur, Nepal	1^st^	34.11	[29]
	**2** ^**nd**^	**55**	
	3^rd^	10.89	

Karnataka, India	1^st^	16	[40]
	**2** ^**nd**^	**54**	
	3^rd^	30	

Kenya	1^st^	10.63	[31]
	2^nd^	21.28	
	**3** ^**rd**^	**68.09**	

North-west, Nigeria	1^st^	0.69	[37]
	2^nd^	6.94	
	**3** ^**rd**^	**52.7**	

Sana'a, Yemen	**1** ^**st**^	**61.1**	[38]
	2^nd^	46.2	
	3^rd^	50	

Southwestern Nigeria	1^st^	2.1	[41]
	2^nd^	37.5	
	**3** ^**rd**^	**92.1**	

Data has been collected from previously reported literature and organized alphabetically.

## Abbreviations

VVC: Vulvovaginal candidosis
NAC: Non-*albicans Candida*
DM: Diabetes mellitus
pH: Potential of hydrogen
HIV: Human immunodeficiency virus
IUD: Intrauterine device.
